# Development of Machine Learning Models for Ion-Selective Electrode Cation Sensor Design

**DOI:** 10.1021/acsestengg.4c00087

**Published:** 2024-03-25

**Authors:** Yuankai Huang, Shifa Zhong, Lan Gan, Yongsheng Chen

**Affiliations:** † School of Civil and Environmental Engineering, Georgia Institute of Technology, Atlanta, Georgia 30332, United States; ‡ Department of Environmental Science, School of Ecological and Environmental Sciences, East China Normal University, Shanghai 200241, China

**Keywords:** ion-selective electrode, sensor, machine learning, Morgan fingerprint, Bayesian optimization

## Abstract

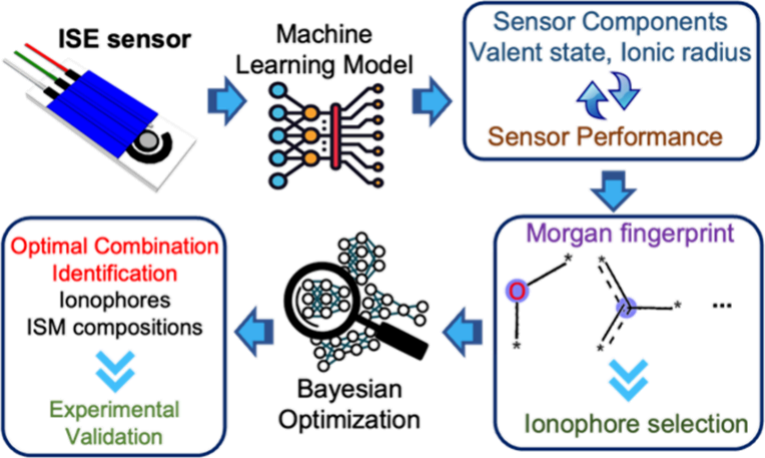

Polyvinyl chloride (PVC) membrane-based ion-selective electrode (ISE) sensors are common tools for water assessments, but their development relies on time-consuming and costly experimental investigations. To address this challenge, this study combines machine learning (ML), Morgan fingerprint, and Bayesian optimization technologies with experimental results to develop high-performance PVC-based ISE cation sensors. By using 1745 data sets collected from 20 years of literature, appropriate ML models are trained to enable accurate prediction and a deep understanding of the relationship between ISE components and sensor performance (*R*^2^ = 0.75). Rapid ionophore screening is achieved using the Morgan fingerprint based on atomic groups derived from ML model interpretation. Bayesian optimization is then applied to identify optimal combinations of ISE materials with the potential to deliver desirable ISE sensor performance. Na^+^, Mg^2+^, and Al^3+^ sensors fabricated from Bayesian optimization results exhibit excellent Nernst slopes with less than 8.2% deviation from the ideal value and superb detection limits at 10^–7^ M level based on experimental validation results. This approach can potentially transform sensor development into a more time-efficient, cost-effective, and rational design process, guided by ML-based techniques.

## Introduction

Water quality sensors are crucial for the functioning of water systems, as they play a vital role in protecting, controlling, and monitoring water treatment processes, detecting anomalies, and assessing performance.^1^ Among different water quality sensors, polymeric membranes-based ion-selective electrode (ISE) sensors have evolved into well-established water quality assessment tools with a detection capability of more than 60 analytes.^2^ Conventional ISE sensor designs rely on a specific affinity for targeting ions to the ionophore in the polymetric membrane matrix.^3^ Therefore, ISE sensor performance (measuring sensitivity and detection limits) relies heavily on the inner structure of the membrane matrix. For instance, the physicochemical properties of different plasticizers can affect the distribution of ionophores inside the membrane, resulting in different ionophore–primary ion complex formations, and finally changing the analytical performance.^4^ To optimize sensing performance, conventional approaches to membrane material design involve a complex, multifaceted process. This process includes selecting suitable membrane materials and fine-tuning the membrane components within a vast, though chemically and practically bounded, space of possibilities. Current studies of ISE fabrication rely on exhaustive experimental investigations by conventional screening procedures. The final analyses can range from an educated guess to empirical observation, resulting in heavy development costs and time loss.^5^ Therefore, precisely identifying optimal combinations of membrane contents is essential to achieving a superior ISE sensor performance.

The past decade has witnessed a rapid growth of machine learning (ML) algorithms in managing complex, multidimensional data sets with low dependence on prior knowledge due to their powerful fitting abilities.^6^ With ML models, sensor performance can be accurately predicted by establishing complex nonlinear relationships between sensor components, sensor structure, and sensor characterization results.^7^ However, previous studies have primarily focused on developing ML models solely based on sensor patterns and structural design. For example, ML models have been applied to assist in the fabrication of nanotube micropatterns^8^ and in determining the geometrical dimensions of pressure sensors.^9^ Much less work has been done in developing ML models based on fabrication conditions and sensor components. In addition to fabrication conditions, the molecular structure and chemistry of membrane components also play critical roles in ISE sensors’ performance.^10^ Thus, another challenge in developing satisfactory ML models is comprehensively describing the membrane components for ISE sensor fabrication. The Morgan fingerprint is one of the most straightforward representations of molecular structure and is capable of capturing trends observed in experimental data.^11,12^ Hence, including the Morgan fingerprint in the model enables the efficient discovery of advanced membrane materials for next-generation ISE sensors.

Despite the growing acceptance of ML models in sensor fabrication, the sole function of ML models developed to date is to predict sensor performance.^13,14^ These ML models cannot guide sensor design because they are unable to identify optimal combinations of membrane components and fabrication conditions from infinite space. Bayesian optimization has recently been adopted in the chemical community and includes automatic chemical design, high-throughput virtual screening, and chemical reaction optimization.^15,16^ Bayesian optimization is designed to balance exploring areas of uncertainty and exploiting available information, leading to a higher quality of configurations in fewer evaluations. However, the most important aspect of Bayesian optimization is its application to diverse search spaces that include arbitrary parametrized reaction domains and its ability to select multiple experiments performed in parallel. Accordingly, the Bayesian optimization approach is well suited to the optimization of the sensor fabrication process. Recently, we successfully applied Bayesian optimization in membrane synthesis optimization to augment water/salt selectivity and permeability.^12^ We were able to predict 10 high-performance monomers capable of delivering an upper-bound-breaking membrane filtration performance. This study is a preliminary demonstration of potential innovation in applied materials science. While the use of metaheuristics for optimal condition design is prevalent in various fields,^17^ our application of Bayesian optimization represents a novel approach, which exemplifies the broader trend of leveraging sophisticated computational techniques to enhance the efficiency and effectiveness of environmental sensor design.

**Figure 1:**
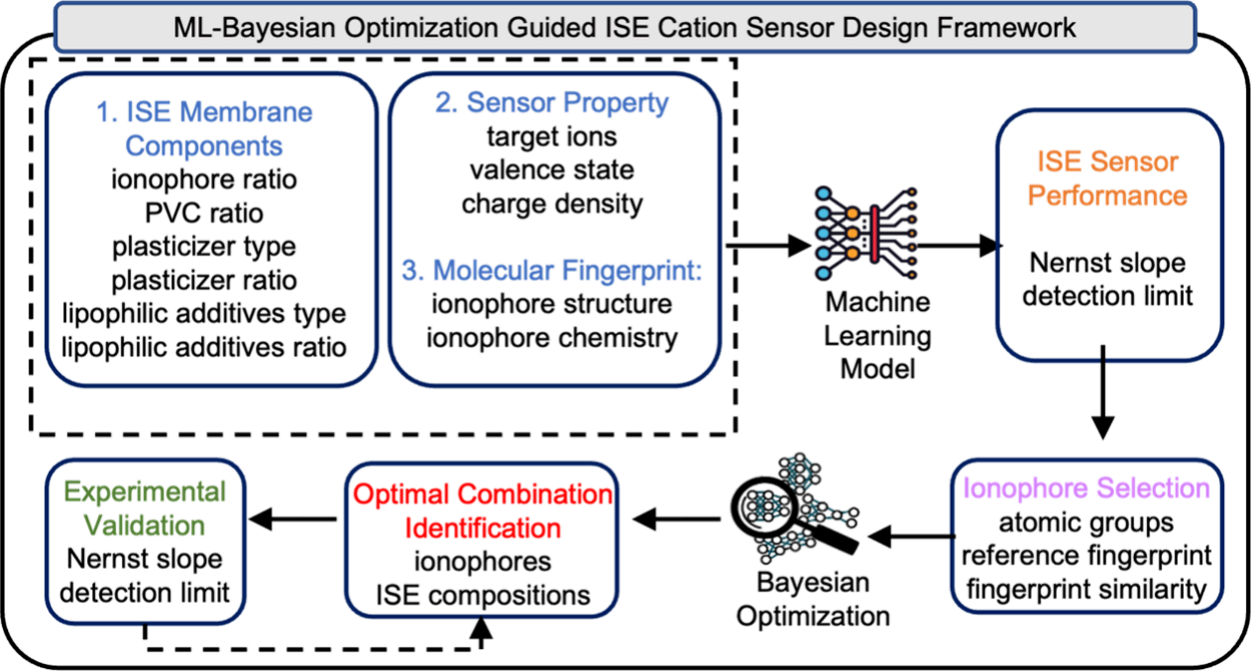
Diagram of the ML-Bayesian optimization framework for ion-selective electrode cation sensor design.

The primary goal of this study is to combine ML, Morgan fingerprint, Bayesian optimization technologies, and experimental results to develop transformative, polyvinyl chloride (PVC) membrane-based ISE cation sensors with exceptional performance, including the Nernst slope and detection limit. We first collected the data sets, including each component in the membrane matrix and the related ISE sensor performances based on published literature from the last 20 years. We then developed appropriate ML models trained with literature-based data sets to enable accurate prediction and a deep understanding of the relationship between ISE components and sensor performance. After the ML models were developed, we then interpreted our model using the Morgan fingerprint and Shapley Additive exPlanation (SHAP) method. This method allowed us to utilize membrane components and ionophore atomic groups with positive contributions toward ISE sensor performance. Furthermore, we applied Bayesian optimization to identify optimal combinations of membrane materials with the potential to deliver a desirable ISE sensor performance. Finally, the experimental validation and performance evaluation of the optimized ISE cations sensors were performed (Figure 1).

## Experimental Section

### Data Set Collection and Construction

To ensure a sufficient data set to develop reliable ML models, we first mined all the data for any membrane components that can affect the PVC membrane-based ISE cation sensor performance from 166 reports published in the last 20 years. The variability in experimental conditions and setups across different studies can introduce a degree of unpredictability into the data set. However, despite these variations, our analysis has identified consistent and genuine correlations within the data, such as between synthesis conditions and material performance. While these differences in experimental setups do influence the overall model performance, they do not negate the existence of underlying patterns that lend validity to our data set. The complete data sets can be found in the Supporting Information. Data for membrane components are divided into two groups: category features (plasticizer type, lipophilic additives type) and numerical features (ionic valence state, ionic radius, PVC ratios, plasticizer ratios, lipophilic additive ratios, and ionophore types, represented by the simplified molecular-input line-entry system, SMILES^18^). The description of input parameters can be found in Table S1. The plasticizer-free data points containing 0% plasticizer and > 80% PVC were deleted since the plasticizer plays a pivotal role in prolonging the ISE sensor lifetime.^19^ Based on the literature research, 242 monovalent cation sensors, 915 divalent cation sensors, and 588 trivalent cation sensor data sets were applied in the ML model development. We then constructed two data sets including (1) Nernst slope (S) and (2) detection limit (L). Not all listed data sets were reported in each paper, and we treated unreported features as missing values. The total number of data points for the two data sets includes 1745 for the S data set and 719 for the L data set (Table 1). The numerical features of these two data sets are summarized in Table S2, including the number of data points and the statistical feature information.

**Table 1:** Total Number of Data Points for the Nernst Slope (S) and Detection Limit (L)

ionic valent state	data set	S data set	L data set
+1	242	242	143
+2	915	915	398
+3	588	588	178
total	1745	1745	719

### ML Model Development and Interpretation

Each data set was randomly partitioned into a training set and a test set using an 80:20 split, where the training set was designated for model construction and the test set for assessing the model’s predictive accuracy. However, this may lead to data leakage because the same ionophore type may exist in both the training and test sets. To prevent this potential data leakage, we also split the data set based on the ionophore type to ensure that the same ionophore type only exists in the training or the test sets. Hence, we developed two types of models: one is with data leakage, while the other is without data leakage.

A 5-fold cross-validation was implemented on the training set, serving to discern the optimal ML algorithm and encoder method and also for adjusting model hyperparameters. To translate categorical values into numerical ones, eight potential encoders, as outlined in the previous literature, were examined. While computational speed varies among different tree-based ML methods, our focus in choosing these algorithms was on their effectiveness in handling overfitting scenarios rather than computation speed alone. Therefore, CatBoost and XGBoost were selected as the ML algorithms of choice, given their superior capability to manage overfitting.^20^ It is noteworthy that both CatBoost and XGBoost are equipped to handle missing data points.^21,22^ Every ML algorithm was paired with each encoder to create models on the training set using 5-fold cross-validation. The optimal ML algorithm and encoder were determined based on their superior cross-validation performance, as gauged by the root-mean-squared error (RMSE)^23^ and *R*^2^ values.
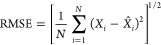
1
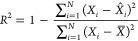
2where *X*_*i*_ and *X̂*_*i*_ are the experimental and predicted values (mV/dec for the Nernst slope and [*M*] for the detection limit). To comprehensively evaluate model performance, we considered both metrics: when models presented comparable RMSE values, their performance was further differentiated based on *R*^2^ values, and similarly, if *R*^2^ values were close, RMSE served as a critical discriminant. This dual-criteria approach ensures a more balanced and thorough assessment of the model’s predictive capabilities.

The type of ionophore, represented by its SMILES notation, was converted to a Morgan fingerprint using the RDKit package in Python. The length and radius of the Morgan fingerprint were defined through Bayesian optimization in the hyperparameter tuning stage. Relevant hyperparameters for the ML models are detailed in Table S3, with a specific emphasis on those that influence the overfitting trend. The ideal radius and length of the Morgan fingerprint, alongside optimal model hyperparameters, were selected based on their ability to achieve the best cross-validation results. Subsequently, the model was trained on the complete training set, foregoing further 5-fold cross-validation, and the model’s performance was evaluated on the test set.

We then merged the data sets to create a larger, all-encompassing one and added a new numerical feature called “Charge” to indicate the charge of the ions since the different ionic charges represent different Nernst slope values.^24^ Here, we treated “Charge” as a numerical feature rather than a categorical feature because the size of the charge property indeed affected the Nernst slope values. The process for developing and evaluating the unified models was the same as that for individual models, and we compared the predictive performance of the unified model with that of the individual models. To interpret our ML models, we used the SHapley additive explanation (SHAP) method. The functioning of SHAP is well documented in previous literature.^25,26^ In general, a feature’s SHapley value measures its contribution as either positive or negative. A feature with a higher absolute SHapley value indicates a more substantial impact on the performance of the ISE sensor.

In addressing the challenge of a low sample-feature ratio, where the feature count exceeds the sample size, we employed advanced algorithms like XGBoost and CatBoost that inherently perform feature selection through hyperparameter tuning.^27^ This approach effectively mitigates the 'curse of dimensionality’ by selectively utilizing features, as evidenced in our updated SHAP plot, where many features demonstrate zero contribution to the model predictions, thereby reducing the effective number of features well below the initial count. However, we still applied feature selection to reduce the number of features. During the hyperparameter tuning process, after generating MF and encoding the categorical features, we removed the features with single values and then calculated the Pearson correlation criterion between two features, and when two descriptors had a correlation exceeding 0.9, one was arbitrarily removed.

### Bayesian Optimization on Synthesis Conditions

After the final ML models were obtained, the selected ionophores based on Morgan fingerprint similarity served as the beginning of ionophore space for Bayesian optimization. We then executed a Bayesian optimization to conclude the optimal synthesis conditions that can deliver the sensors with desirable performance. In our study, Bayesian optimization was conducted using the hyperopt package in Python, with the Tree of Parzen Estimators (TPE) algorithm serving as the primary tool. The TPE algorithm is particularly suited to our needs, as it effectively models the probability distribution of the condition space based on the objective function values. For each feature, we first determined their range to set up a space for further exploration (Table 2). The most influential parameters and membrane components determined by the ML model interpretation included ionic valent states, ionic radius, PVC ratio (range: 20–66 wt%), plasticizer ratio (range: 20–73 wt%), ionophore ratio (range: 0–18.5 wt%), additives ratio (range: 0–19 wt%), plasticizer types (text values), and additive types (text values), which were reasonably defined according to the literature-based data sets (Tables 2 and S2). All of the defined ranges for membrane components are unified with the selected ionophores to form a space for Bayesian optimization. Then, we applied Bayesian optimization by using the hyperopt package to explore the space to propose the optimal combination of synthesis conditions for the best sensor performance (slope = 59.16 mV/dec for monovalent sensors, 29.58 mV/dec for divalent sensors, and 19.72 mV/dec for trivalent sensors based on the Nernst equation). All of these variables in the space were together optimized during Bayesian optimization.

**Table 2:** Range of Each Feature Applied to Bayesian Optimization

features	ranges (wt%)
PVC ratio	20–66
plasticizer ratio	20–73
ionophore ratio	0–18.5
additive ratios	0–19

### ISE Sensor Fabrication and Performance Evaluation

Two of each representative ISE monovalent, divalent, and trivalent cation sensor found in training/test data sets were fabricated to verify ML predictions. The monovalent ions include Li^+^ (6,6-dibenzyl-14-crown-4 as ionophore^28^) and NH_4_^+^ (nonactin as ionophore^29^). The divalent ions include Cu^2+^ (*o*-Xylylenebis(*N*,*N*-diisobutyldithiocarbamate as ionophore^30^) and Ca^2+^ (Calimycin as ionophore^31^). The trivalent ions include La^3+^ (*N*′-(1-Pyridin-2-ylmethylene)-2-furohydrazide as ionophore^32^) and Tm^3+^ (1-(2-thiazolylazo)-2-naphthol as ionophore^33^). Their use in verifying ML model predictions was based on sensor performance. Each type of sensor contains five different recipes (i.e., 30 recipes in total), and the details of the membrane components are described in Table S4. A good agreement of our experimental results with the predicted results from ML models validated the reliability and prediction accuracy of our ML models. Then, using the same fabrication procedure, three different ISE cation sensors (the sodium ion (Na^+^), also referred to as a sodium cation, the magnesium ion (Mg^2+^), a divalent metal cation, and the aluminum ion (Al^3+^), also a cation), were fabricated according to the optimized fabrication combinations identified by Bayesian optimization (Table S5). The details of the ISE sensor fabrication and characterization processes were described previously^34,35^ and can be found in Text S1.

## Results and Discussion

### Comparison of Model Performances

Table 3 summarizes the model performances on training and test sets in terms of the RMSE and coefficient of determination (*R*^2^). For individual models, the *R*^2^ on the test sets increased as the ionic valent state increased, as indicated by 0.32 in monovalent sensors, 0.43 in divalent sensors, and 0.61 in trivalent sensors (Table 3, Figure S1). After merging the data sets to develop a unified model, the *R*^2^ substantially improved to 0.75. Using this model to predict each individual test set, we found that the corresponding *R*^2^ for each ionic valent state also improved compared to the individual ML models (Table 3). This result indicates that merging a similar data set to create a large one is beneficial to building a more robust ML model due to the improved generalization and avoiding overfitting, which is consistent with our recent findings.^12^ After feature selection, however, we observed a slight decrease in the test performance (Table 3).

**Table 3:** Evaluation of Model Performance in the Nernst Slope

data set		train. size	train. *R*^2^	train. RMSE	test size	test *R*^2^	test RMSE	exp. size	exp. *R*^2^	exp. RMSE
with data leakage										
individual	+1	193	0.86	4.40	49	0.32	11.31	5		
	+2	704	0.70	4.23	176	0.43	6.58	5		
	+3	470	0.86	2.74	117	0.61	4.54	5		
merged	+1				49	0.42	10.42			
	+2				176	0.46	6.38			
	+3				117	0.63	4.44			
	together	1367	0.93	3.38	342	0.75	6.58	15	0.86	5.10
	together^a^	1367	0.93	3.49	342	0.74	6.76			
without data leakage										
individual	+1	202	0.31	10.16	40	0.10	11.73	5		
	+2	709	0.48	5.95	171	0.19	5.61	5		
	+3	451	0.25	6.25	137	0.06	8.79	5		
merged	+1					0.24	10.82			
	+2					0.14	5.75			
	+3					0.49	6.45			
	together	1362	0.85	5.09	348	0.70	6.79	15	0.93	3.60

a Applying feature selection.

However, literature-based data can naturally have data leakage concerns where shared information across data from the same source can lead to overly optimistic results. To address this issue, it is crucial to adopt a more strategic approach to data splitting. In our study, we redeveloped our models using the new data-splitting approach based on previous reports,^36,37^ and the revised model performance metrics are presented in Table 3. Comparing the results with potential data leakage, after preventing data leakage, we observed a notable decrease in the performance metrics for individual models (Table 3). This change highlights the impact of data leakage on model accuracy. However, the performance of the ‘Merged’ models showed only a slight decline (test *R*^2^ = 0.70, RMSE = 6.79 compared to test *R*^2^ = 0.75, RMSE = 6.58 for the model with data leakage), indicating robustness even when data leakage is controlled (Table 3).

Compared to the Nernst slope, the ML models did not correlate well with the relationship between the detection limit and synthesis conditions. The *R*^2^ values on the test sets were not satisfactory, as demonstrated by 0.29 in monovalent sensors, 0.10 in divalent sensors, and 0.09 in trivalent sensors. Even merging data sets did not work, and the unified model only achieved 0.29 of *R*^2^ (Figure S2, Table S6). Since monovalent cations tend to have more stable and commercialized ISE sensors,^5,38^ few studies have focused on the optimization of the membrane compositions for detection limits (141 test datapoints), leading to the low *R*^2^ value coming from data sparsity from previous reports. Additionally, previous reports’ detection limits were calculated as the intersection of the two slope lines at low-concentration ranges and high-concentration ranges (Figure S3), leading to a weak predictive performance in ML model development.^39,40^

In addition to validating the predictive accuracy of our built models based on test data sets, we also performed experimental validation by testing 30 fabricated PVC membrane-based ISE sensors (Table S4). We found that experimental results for the Nernst slope showed relatively good agreement with the predicted values (*R*^2^ and RMSE values were 0.86 and 5.10, respectively), supporting the reliability of our built models. Moreover, we observed a significant difference in performance metrics when we addressed the issue of data leakage. The model developed without data leakage demonstrated markedly improved performance, with *R*^2^ and RMSE values of 0.93 and 3.60, respectively. This notable enhancement underscores the critical importance of effectively managing data leakage in material-related data set analyses, as it substantially impacts the accuracy and reliability of predictive models. This finding indicates that the application of the ML method enables the investigation of ISE sensor components not previously reported in the literature. Moreover, it provides strong evidence ensuring the feasibility of the Morgan fingerprinting method. Since the ML model detection limits could not be used for further optimization, the optimal detection limit was determined by the experimental results performed according to the Nernst slope optimization.

### Interpretation of the ML Model

Following the validation of the ML model for the Nernst slope, we then interpreted the model to understand the mechanism of prediction from the ionophore structures and membrane components. This understanding is essential in the evaluation of ML model predictions and ensures consistency with fundamental domain knowledge and experimental experience.

**Figure 2:**
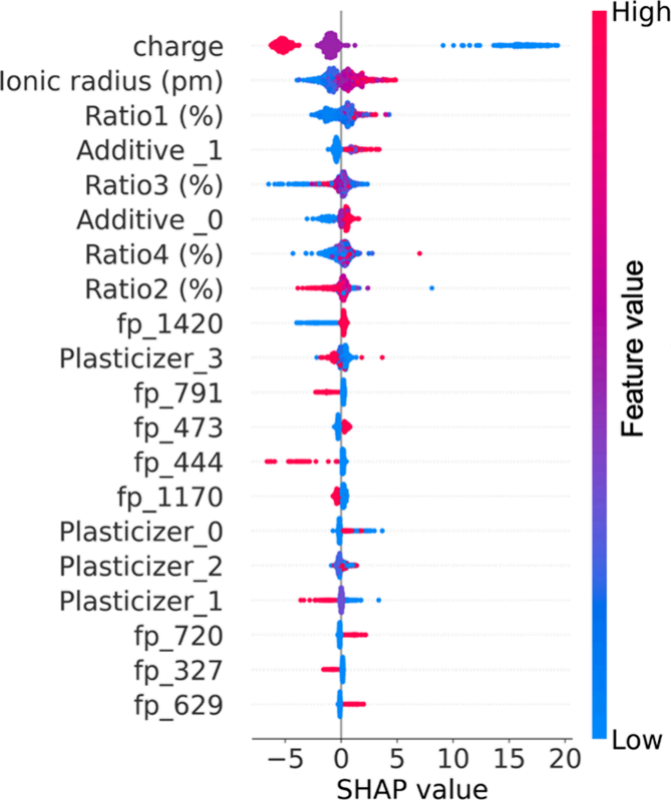
Shapley additive explanation (SHAP) plot interprets the models’ fabrication condition contributions to the Nernst slope in the data set. The *X*-axis is the SHAP value, and the positive values indicate that the Nernst slope can be increased by the specific features listed on the left *Y*-axis. Meanwhile, the negative values indicate a reduction in the Nernst slope. The size of each feature’s value is colored from blue to red, corresponding to the smallest and largest values (in the charge states, blue is +1, purple is +2, and red is +3). The pattern for each feature is composed of small dots, and each dot represents a sample containing this feature.

From the Shapley values, calculations were based on the Nernst slope. The most critical contributions were ionic charge states (a negative contribution), ionic radius (a positive contribution), the PVC ratio (ratio 1, a positive contribution), the ionophore ratio (ratio 3, which was not an obvious trend), the lipophilic additive ratio (ratio 4, not an obvious trend), and the plasticizer ratio (ratio 2, a negative contribution) (Figure 2). These results show agreement with the widely recognized knowledge of ISE sensor properties. The Nernst slope of the ISE sensors is highly dependent on the charge state of the target ions. Thus, the Nernst slope decreases as the ionic valent state increases. Meanwhile, the ionic radius decreases as shown in our Table S2 data set. We did not further analyze the effects of the categorical features (plasticizer type and lipophilic additives type) because they were encoded to a different number of new features rather than a single feature. The Shapley value trend shown in each new feature did not determine or indicate whether a plasticizer type was good or not.

In addition to the charge state and ionic radius, the performance of ISE sensors depends significantly on membrane components (e.g., PVC/plasticizer ratio, ionophore ratio, and lipophilic additives ratio), which can affect the mobility of the target cations in the inner polymeric chain and the morphology of ISE, as described by the Nikolskii–Eisenman Formalism.^41^ Typically, these properties are tuned by altering membrane components, consistent with those identified by Shapley values from the perspective of positive or negative contributions. For the PVC ratio, it is well-established that besides the ionophore ratio, the PVC membrane-based cation ISE sensors exhibit a Nernstian response owing to the fortuitous presence of anionic impurities in the PVC matrix.^42^ When the ionophore is present in the membrane matrix, the PVC ratio shows a positive contribution to the Nernst slope and will provide even results in a super Nernst slope response (i.e., the Nernst slope is higher than the ideal value).^43^ A proper plasticizer/PVC ratio (e.g., 2:1) is essential during the membrane fabrication process. This is because the plasticizer ratio can decrease the glass transition temperature below the PVC ambient temperature and thereby maintain the membrane’s physical and mechanical properties.^19^ However, in PVC membrane-based ISE cation sensors, increasing the plasticizer ratio can cause poor adhesion of the membrane due to reduced peel-off forces caused by the liquid film formation between the membrane and water.^44^ This liquid film not only deteriorates the sensor’s lifetime but also poses a negative contribution to the Nernst slope by blocking the cation diffusion rate and causing anion interference.^45^ For the ionophore and lipophilic additive ratios, increasing both components to establish a proper ratio in the membrane is mandatory. The ratio improves the Nernst slope performance, which provides neutral carriers and anionic sites for specific ion diffusion.^46,47^ However, a decreased Nernst slope has been observed when the ratio of ionophores or additives is too high due to nonspecific ion exchange and coion interference.^48^ The association of SHapley Additive exPlanation (SHAP) values with the underlying ISE mechanisms controlled sensor performance enhanced the reliability of our built models.

### Ionophore Effects on Sensor Performance

**Figure 3:**
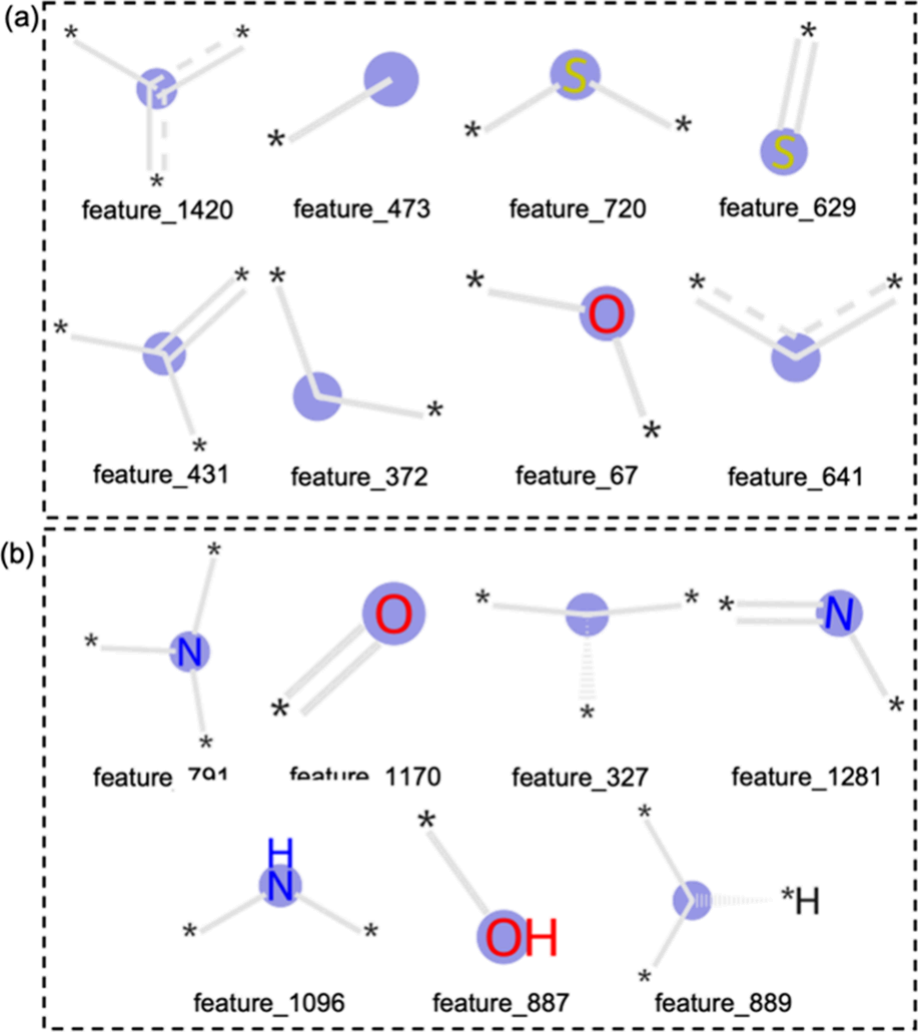
Atomic groups serve as (a) positive contributors and (b) negative contributors to the Nernst slope. The unlabeled blue circles represent carbon atoms. Each feature number denotes the feature position in the Morgan fingerprint vector. The blue circles are atoms. The gray lines represent the bonds that are not included in the features. The asterisks (*) represent other atoms that are connected.

Based on the SHAP value of each Morgan fingerprint (Figure S4), eight atomic groups with the most positive contribution and seven atomic groups with the most negative contribution to the Nernst slope were determined. As shown in Figure 3, the atomic group feature_1420, feature_473, feature_720, feature_629, feature_431, feature_372, feature_67, and feature_641 have the most positive contributions to the Nernst slope.

This agrees with previous research establishing the role atomic groups with a neutral charge and hydrophobic properties play in providing essential lipophilic sites of opposite charge for optimum Nernst slope performance.^49^ Additionally, the atomic groups tend to form hydrogen bonds with the analyte (e.g., feature_720, feature_372, and feature_67) which are essential to the formation of the ionophore complex with target ions thereby making a positive contribution to the Nernst slope.^50^ By contrast, the negative contribution of feature_791 and feature_1170 may come with the extensively existing tertiary amines and carbonyl groups which can only function as hydrogen-bond acceptors.^48^ These results suggest that hydrogen bonds may have a larger contribution to capturing cations than the cation–dipole interaction for currently studied ionophores. Additionally, the atomic groups with nitrogen atoms (feature_791, feature_1281, and feature_1096) can be protonated under the working pH range of the ISE sensors, which hinders the capture of cations by charge repulsion in ISE sensors.^51^ Further, the atomic groups with hydrophilic properties (feature_791, feature_1170, feature_1281, feature_1096, and feature_887) reserve an undesired thin water layer between the ionophore and the underlying electrode, destabilizing the electrical potential response and finally giving a negative contribution to the Nernst slope performance.^52,53^

### Bayesian Optimization To Identify Optimal Combinations and Experimental Validation

To determine the optimal membrane components for the best sensor performance, we first determined the candidate ionophores for evaluation. Besides the 195 ionophores we collected from previous reports, we also added 23 new ionophores from the National Library of Medicine (NLM) database to screen (Table S7). These ionophores have been synthesized to date but are not included in our data set. We then defined the combination space and initialized reasonable ranges for different membrane components (Table S2). Bayesian optimization offers the opportunity to identify the optimal combinations of ionophores and membrane components. These ionophore and membrane component combinations enable the fabrication of ISE sensors with ideal Nernst slopes.

**Figure 4:**
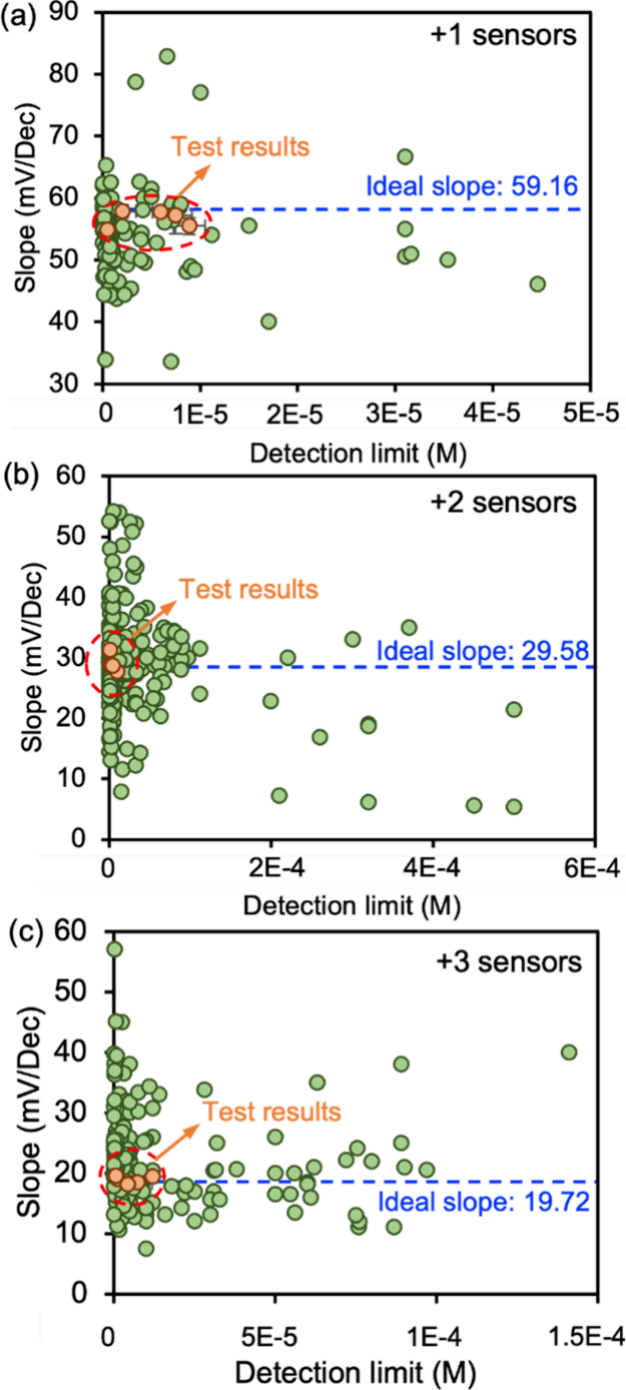
Identification of optimal combinations from Bayesian optimization.

Five of each membrane recipe for sodium ion (Na^+^), manganese ion (Mg^2+^), and aluminum ion (Al^3+^) sensors were based on the commercially available ionophores chosen from the 16 candidates. They were simulated to achieve the ideal Nernst slope (i.e., 59.16 mV per charge for Na^+^, 29.58 mV per charge for Mg^2+^, and 19.72 mV per charge for Al^3+^) (Table S8). Table S5 lists the plasticizer/PVC ratios ranging from 0.3 to 1.8 based on the different types of plasticizers provided by Bayesian optimization. The plasticizer ratio strongly affects the Nernst slope performance of the ISE sensors due to its influence on dielectric constants (ε^r^), the mobility of the ionophore molecules, and the state of ligands inside the membrane matrix.^54^ A sufficient amount of plasticizer is critical to facilitating the diffusion of the target ion–ionophore complex inside the membrane matrix.^19^ Nevertheless, a continuous increase in the ratio of plasticizers may lead to subpar miscibility between the plasticizers and PVC. This could potentially induce poor adhesion between the membrane and electrode, possibly causing substantial leakage of the ionophore and lipophilic additives from the membrane matrix.^19,55^ It has been reported that the sensor accuracy and Nernst slope could be diminished at high plasticizer ratios.^56^ This has also been verified by the SHAP values calculated in Figure 2. Based on our optimal membrane components, a relatively lower ratio of plasticizer/PVC was obtained compared to previous research (1.5–2).^57^ For the ionophore and lipophilic additive, modest numbers of anionic sites provided by the lipophilic additives have improved the potentiometric properties of ISE sensors, but a low ratio of ionophore/lipophilic additives (usually < 0.5) can substantially decrease the Nernst slope due to nonspecific ion exchange through the formation of ion pairs.^48^ However, from the optimal membrane components results of Na^+^ and Mg^2+^ sensors, the presence of negative contribution atomic groups has been obvious—such as feature_791, feature 1170, and feature 1096 in the selected ionophores (Figure 4b and Table S5), resulting in the lower ratios of ionophore/lipophilic additives (0.15–0.45) in the Bayesian optimization results compared to previous reports.^58^ Even though these atomic groups had a negative effect on the Nernst slope, they are critical to forming the cyclic cavities/semicavities to help the ionophore serve as ion carriers.^59^ The presence of positive atomic groups such as feature_720, feature_629, feature_372, and feature_67 served as hydrogen bond donors in these selected ionophores. In the optimal membrane components result of the Al^3+^ sensor, due to a large number of positive contribution atomic groups present in the selected ionosphere (feature_372), the ratios of ionophore/lipophilic additives were higher than the Na^+^ and Mg^2+^ sensors (4–6) to achieve the optimal Nernst slope (Table S5).

To validate the Nernst slope predicted results and identify the best sensor recipe to achieve the lowest detection limit, we tested the sensor performance according to Bayesian optimization results for each sensor recipe. The ionophores and the detailed membrane compositions are summarized in Table S5. Experimental results were transformed to the corresponding Nernst slope/detection limit and compared with the existing sensor results from previous reports (Figure 4). All 15 experimental results based on the Bayesian optimization results showed excellent Nernst slope performance with errors < 7.4% in the Na^+^ sensor, < 6.3% in the Mg^2+^ sensor, and < 8.2% in the Al^3+^ sensor compared to the ideal Nernst slope (Figure 4). For the detection limit, some membrane recipes from the Bayesian optimization results showed promising detection limits at the 10^–7^ M level, which are almost the lowest detection limit in our data set. However, some experimental results for the detection limit are not competitive compared to previous research, with the highest value of 8.88 × 10^–6^ M for the Na^+^ sensor, 9.58 × 10^–6^ M for the Mg^2+^ sensor, and 1.21 × 10^–5^ M for the Al^3+^ sensor (Figure 4). Even though a wide range of input features, such as membrane thickness^60^ and membrane surface area^61^ can affect sensor performance, the candidate fabrication conditions used for Bayesian optimization were restricted to a smaller space since the membrane morphology is difficult to control during the ISE membrane fabrication and coating process. We mainly focused our attention on the most effective conditions for sensor performance, as highlighted by SHAP values (Figure 2). Therefore, aside from the reason that no desirable ML models are applicable to detection limit predictions, the model may show a weaker predictive performance in this smaller space. Moreover, when developing ML models, the solid contact layer was not included as an input feature. However, the solid contact layer effect on the detection limit might be another reason for the discrepancy.^2^ Thus, the future candidate input features used in the Bayesian optimization could be extended to include the material type in the solid contact, the solid contact thickness, and membrane/solid contact morphology, as well as the most effective fabrication conditions mentioned above.

## Environmental Impacts and Future Perspective

In this study, we propose a transformative, data-driven computational framework for the development of PVC membrane-based cations in ISE sensors. We constructed a reference Morgan fingerprint based on the chosen atomic groups derived from ML model interpretation, allowing the rapid screening of ionophores. By identifying the optimal combinations of ionophore materials and membrane components, Bayesian optimization allowed us to fabricate PVC membrane-based cation ISE sensors with ideal Nernst slopes and low detection limits. The fabricated Na^+^, Mg^2+^, and Al^3+^ sensors based on the Bayesian optimization results exhibited excellent Nernst slopes with deviations < 8.2% from the ideal value among all the predicted sensors and a superb detection limit at the 10^–7^ M level among part of the experimental results. Compared to our previous membrane-design research,^12^ the ML-assisted ISE sensor design presents significant scientific advancements. In particular, the utilization of Shapley values and Morgan fingerprint results provides a more profound comprehension of the relationships between membrane components and sensor performance. By scrutinizing crucial features and their contributions to sensor performance, researchers can identify previously unknown structure–performance relationships, ultimately resulting in the creation of more efficient and innovative ISE sensors. Furthermore, ML models trained on diverse data sets from this study are versatile and can be adapted to various sensing applications such as fluorescent ion-sensing arrays design^62^ and biological cell membranes.^63^ Such groundbreaking results provide enormous potential for data-driven sensor design.

We believe that the successful execution of this project will potentially shift the paradigm of the conventional experiment-driven sensor development process to a more time-efficient, cost-effective, and rational membrane design strategy guided by ML-based techniques. Advances in data-driven methods powered by rapidly developed computing resources will significantly reduce the time and cost previously devoted to screening materials, thereby enhancing research efficiency. The rich database accumulated over time, including materials design, ISE sensor fabrication parameters, and performance, will be beneficial to future research in other sensor designs. The low-cost ISE sensors fabricated from this project are essential for real-time water quality monitoring. They meet an urgent need for more efficient monitoring technologies that can promptly identify water quality problems, proactively assess wastewater treatment system performance, and execute automated decisions to fix operational problems.^64^

In this work, the authors mainly focused on the conventional PVC membrane-based ISE sensors without considering the impacts of alternative polymer matrices and solid contacts. Therefore, the models developed here cannot be applied to the new generation of ISE sensors, such as PVC-free ISE sensors or sensors with material modifications. Additionally, the *R*^2^ values of ML test results for the detection limit are not desirable for Bayesian optimization, and the sensor selectivity was not considered in this study due to the data sparsity. However, since the prediction performance of the ML models strongly depends on the availability, accuracy, and size of a data set, it will benefit from more studies related to the parameters being published. The strategy demonstrated in this contribution can be easily extended to develop appropriate models and guides in designing different types of ISE sensors for water quality monitoring.^65^

## Supporting Information Available

Details of the ISE sensors fabrication and characterization processes; Nernst slope correlation of experimental results with predicted values; detection limit correlation of training results with test results; example to show how to determine the detection limit in the cation ISE sensors; SHAP plot of features in the Morgan fingerprint for the Nernst slope; description of input and output features for model development; statistical information of the numerical features as model inputs; the range of candidate hyperparameters for each ML algorithm; membrane components and ratios used for ISE sensor fabrication to validate the prediction accuracy of the built models; membrane components and ratios used for ISE sensor fabrication to validate the Bayesian optimization on the Nernst slope and to determine the optimal detection limit; evaluation of model performance in the detection limit; the new candidate ionophores used for Bayesian optimization which are not included in the data set; and 16 candidate ionophores with MF similarities to both reference MFs to execute Bayesian optimization (PDF)
