# Supplementary material for: Development of Machine Learning Models for Ion-Selective Electrode Cation Sensor Design
Source: ACS ES T Eng. 2024 Mar 25;4(7):1702–11. doi: 10.1021/acsestengg.4c00087 (PMC11250033; doi:10.1021/acsestengg.4c00087)
Supplement: Supplementary file 1 — ee4c00087_si_001.pdf [file ee4c00087_si_001.pdf]

Supplementary Information for

# Development of Machine Learning Models for Ion-Selective Electrode Cation Sensor Design

Yuankai Huang [a†], Shifa Zhong [a,b†], Lan Gan [a], Yongsheng Chen [a*]

[a] School of Civil and Environmental Engineering, Georgia Institute of Technology, Atlanta, Georgia 30332, United States

[b] Department of Environmental Science, School of Ecological and Environmental Sciences, East China Normal University, Shanghai 200241, PR China

(*Corresponding author: Yongsheng Chen, Email: yongsheng.chen@ce.gatech.edu, Phone: 4048943089)

[†]These authors contributed equally to this paper.

Number of pages: 18

Number of texts: 1

Number of tables: 8

Number of figures: 4

## Text S1. Details of the ISE Sensors Fabrication and Characterization Processes

The ISE sensors (50 mm length x 13 mm width) were fabricated using screen-printing technology (eDAQ, ET083) with a graphitic carbon working electrode (5 mm diameter disk). The sensor membrane was prepared by drop-casting 10 μL of ionophore polymer cocktails (calculated from the ML and Bayesian optimization results) on the working electrode's surface. Then, the potentiometric measurements were carried out using a BASi PalmSens4 potentiostat (PalmSens BV, Houten, Utretch, The Netherlands) at room temperature with Ag/AgCl (3 M KCl) as the reference electrode. The detection limits (sensitivity) of the sensors were determined by starting with the target ion concentrations of $1 \times 10^{-7}$ M, followed by recording the potential readings in incremental concentrations ($10^{-6.5}$ to $10^{-1}$ M). All the sensor tests were examined in triplicate.

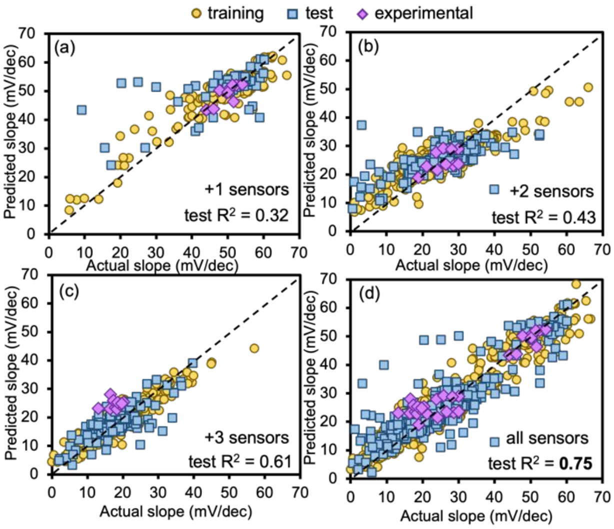


**Fig. S1**. Nernst slope correlation of experimental results with predicted values among (a) monovalent, (b) divalent, (c) trivalent, and (d) all sensors. Yellow dots represent training results; blue dots are test results, and purple dots are experimental results. For experimental validation, we used the monovalent cations, lithium ($Li^+$) and ammonium ($NH_4^+$), the divalent metal cation copper ($Cu^{2+}$) and the divalent mineral cation, calcium ($Ca^{2+}$), as well as the trivalent cation, lanthanum ($La^{3+}$) and trivalent ion, thulium ($Tm^{3+}$). Materials and the corresponding fabrication conditions are summarized in Table S4.

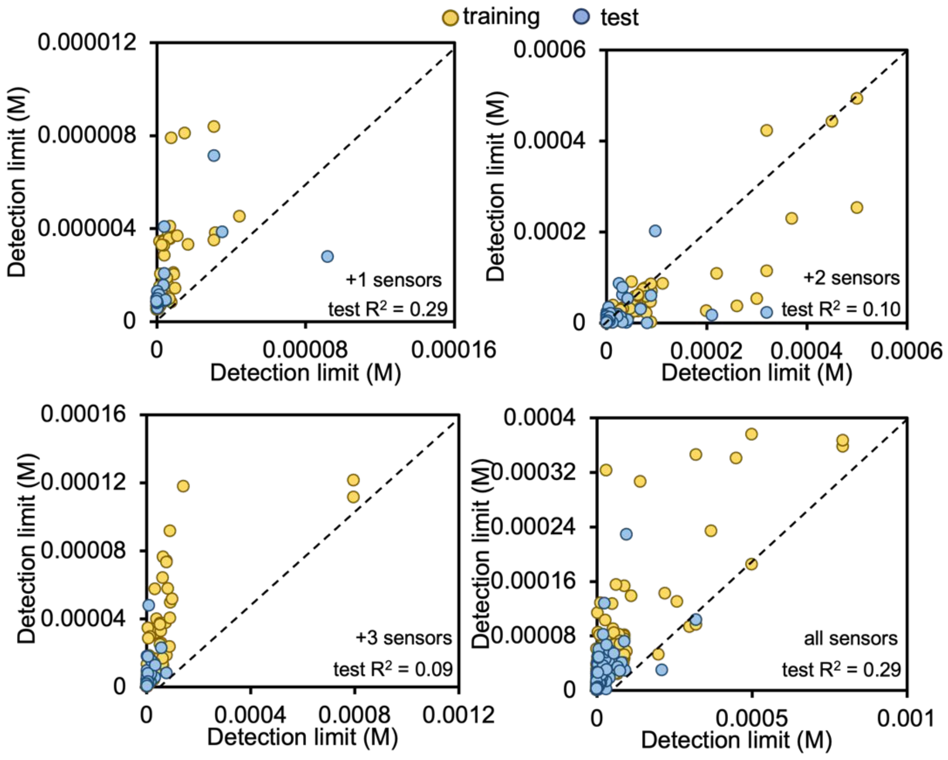


**Fig. S2**. Detection limit correlation of training results with test results among (a) monovalent, (b) divalent, (c) trivalent, and (d) all sensors. Yellow dot: training results, blue dot: test results.

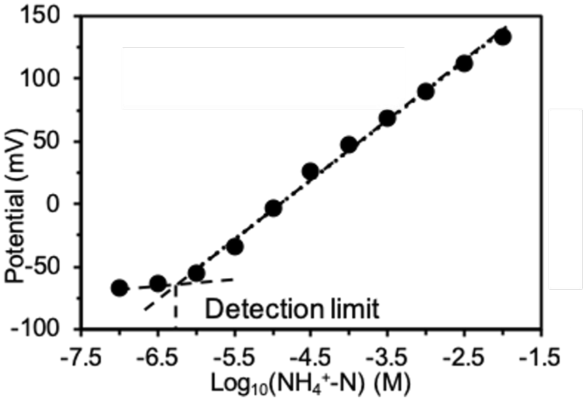


**Fig. S3**. An example to show how to determine the detection limit in the cation ISE sensors.

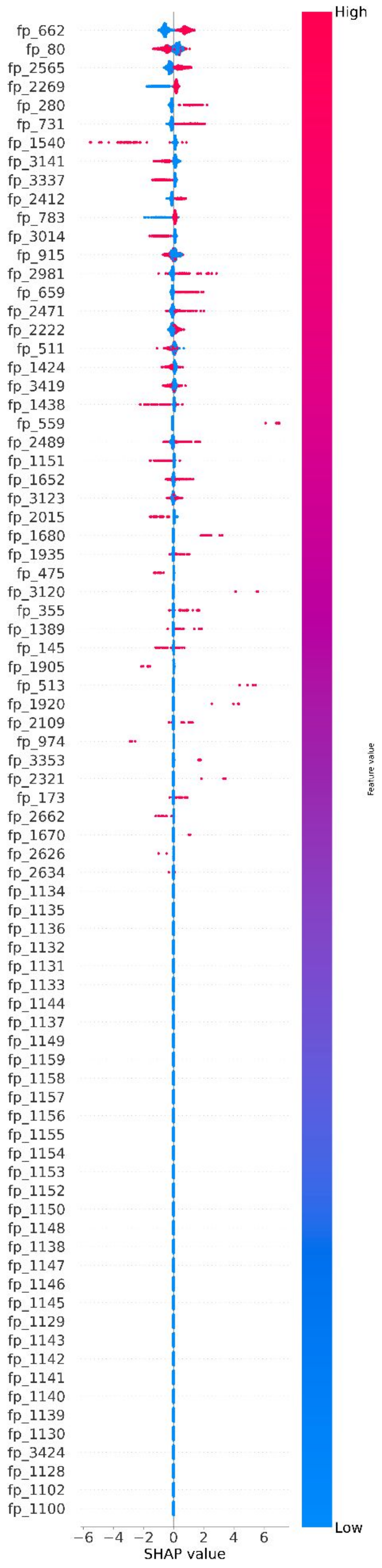
fp_662
fp_80
fp_2565
fp_2269
fp_280
fp_731
fp_1540
fp_3141
fp_3337
fp_2412
fp_783
fp_3014
fp_915
fp_2981
fp_659
fp_2471
fp_2222
fp_511
fp_1424
fp_3419
fp_1438
fp_559
fp_2489
fp_1151
fp_1652
fp_3123
fp_2015
fp_1680
fp_1935
fp_475
fp_3120
fp_355
fp_1389
fp_145
fp_1905
fp_513
fp_1920
fp_2109
fp_974
fp_3353
fp_2321
fp_173
fp_2662
fp_1670
fp_2626
fp_2634
fp_1134
fp_1135
fp_1136
fp_1132
fp_1131
fp_1133
fp_1144
fp_1137
fp_1149
fp_1159
fp_1158
fp_1157
fp_1156
fp_1155
fp_1154
fp_1153
fp_1152
fp_1150
fp_1148
fp_1138
fp_1147
fp_1146
fp_1145
fp_1129
fp_1143
fp_1142
fp_1141
fp_1140
fp_1139
fp_1130
fp_3424
fp_1128
fp_1102
fp_1100
−6 −4 −2 0 2 4 6
SHAP value
High
Low
Feature value

**Fig. S4.** The SHAP plot of features in the Morgan fingerprint for the Nernst slope. (a) positive contribution features in the training dataset (b) negative contribution features in the training dataset. The X-axes are the Shapley values where the positive value means that the Nernst slope can be increased by the specific features, while the negative value indicates a resultant reduction in the Nernst slope. The size of each feature's value is colored from blue to red, corresponding to the smallest and largest values. The pattern for each feature is composed of small dots and each dot represents one sample containing this feature.

**Table S1.** Description of input and output features for model development.

| Category | Parameter | Unit | Description |
|---|---|---|---|
| Text | plasticizer type | / | To maintain the proper physical and mechanical properties of the membrane |
| | lipophilic additives type | / | Improve the binding ability of the membrane |
| Numerical | ionic valence state | / | Different ions show different Nernst response |
| | ionophore type (converted to MF) | / | Selectively binds the target ions |
| | ionic radius | pm | The ionophores are fabricated based on the ionic radius of the target ions |
| | PVC ratio | wt% | To reduce the mobility of the ionophores and additives inside the membrane |
| | plasticizer ratio | wt% | The ratio can change the membrane stability |
| | ionophore ratio | wt% | Affects the sensor's affinity to target ions, changes the sensor sensitivity, selectivity, and lifetime |
| | lipophilic additives ratio | wt% | Affects the sensor's potentiometric response, changes the sensor sensitivity, selectivity, and lifetime |

**Table S2.** Statistical information of the numerical features as model inputs.

| Feature | Count | Mean | Std | Min | 25% | 50% | 75% | Max |
|---|---|---|---|---|---|---|---|---|
| **S dataset** | | | | | | | | |
| ionic valence state | 1745 | 2.20 | 0.66 | 1 | 2 | 2 | 3 | 3 |
| ionic radius | 1745 | 96.04 | 41.53 | 45 | 73 | 95 | 115 | 350 |
| PVC ratio | 1745 | 33.01 | 5.75 | 20 | 30 | 32 | 33 | 66.01 |
| plasticizer ratio | 1744 | 61.51 | 6.27 | 20 | 60 | 63.1 | 65.3 | 72.4 |
| ionophore ratio | 1745 | 3.45 | 2.57 | 0 | 1.2 | 3 | 5 | 18.5 |
| lipophilic additives ratio | 1743 | 2.07 | 2.53 | 0 | 0.28 | 1.26 | 3 | 18.7 |
| **L dataset** | | | | | | | | |
| ionic valence state | 719 | 2.05 | 0.67 | 1 | 2 | 2 | 2 | 3 |
| ionic radius | 719 | 95.59 | 37.30 | 45 | 73 | 95 | 115 | 350 |
| PVC ratio | 719 | 34.48 | 6.03 | 23.4 | 31.5 | 32.8 | 33.9 | 61.67 |
| plasticizer ratio | 719 | 60.96 | 7.04 | 20 | 59.4 | 63.57 | 65.4 | 72.7 |
| ionophore ratio | 719 | 2.97 | 2.34 | 0 | 1.09 | 2.15 | 4.68 | 18.5 |
| lipophilic additives ratio | 719 | 1.65 | 1.84 | 0 | 0.5 | 1.07 | 2 | 10 |

**Table S3**. The range of candidate hyperparameters for each ML algorithm.

| ML algorithm | Range of hyperparameter |
|---|---|
| XGBoost | colsample_bytree: [0, 1],<br>learning_rate: [0, 1],<br>max_depth: [1,6],<br>subsample: [0.0, 1.0],<br>reg_alpha:[0, 10],<br>gamma:[0, 20],<br>reg_lambda:[1, 10],<br>n_estimators: [1, 100],<br>fp_radius:[0, 5],<br>fp_length: [100, 5048] |
| CatBoost | depth: [1,6],<br>l2_leaf_reg: [0,100],<br>iterations: [1,1000],<br>bagging temperature: [1, 200],<br>random strength: [1,200],<br>fp_radius: [0, 5],<br>fp_length: [100, 5048] |

**Table S4.** Membrane components and ratios used for ISE sensor fabrication to validate the prediction accuracy of the built models.

| No. | Target ion | Ionic radius | PVC ratio (%) | Plasticizer type/ratio (%) | Ionophore structure/ratio (%) | Lipophilic additives type/ratio (%) |
|---|---|---|---|---|---|---|
| **monovalent sensors** | | | | | | |
| | | | | 2-Nitrophenyl octyl ether | 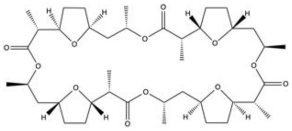 | Sodium tetraphenylborate |
| 1 | | | 33 | 63 | 4 | 0 |
| 2 | | | 35 | 61 | 3.5 | 0.5 |
| 3 | $NH_4^+$ | 154 | 37 | 59 | 3 | 1 |
| 4 | | | 39 | 57 | 2.5 | 1.5 |
| 5 | | | 41 | 55 | 2 | 2 |
| | | | | Dibutyl phthalate | 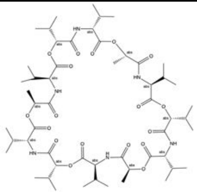 | Potassium tetrakis(4-chlorophenyl)borate |
| 6 | | | 28 | 66 | 6 | 0 |
| 7 | | | 30 | 64 | 5.5 | 0.5 |
| 8 | $Li^+$ | 76 | 32 | 62 | 5 | 1 |
| 9 | | | 34 | 60 | 4.5 | 1.5 |
| 10 | | | 36 | 58 | 4 | 2 |

| No. | Target ion | Ionic radius | PVC ratio (%) | Plasticizer type/ratio (%) | Ionophore structure/ratio (%) | Lipophilic additives type/ratio (%) |
|---|---|---|---|---|---|---|
| **divalent sensors** | | | | | | |
| | | | | Dibutyl phthalate | 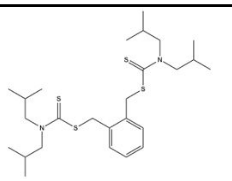 | Sodium tetraphenylborate |
| 1 | $Cu^{2+}$ | 73 | 30 | 66 | 4 | 0 |

| | | | | | | |
|---|---|---|---|---|---|---|
| 2 | | | 32 | 64 | 3.5 | 0.5 |
| 3 | | | 34 | 62 | 3 | 1 |
| 4 | | | 36 | 60 | 2.5 | 1.5 |
| 5 | | | 38 | 58 | 2 | 2 |
| | | | | 2-Nitrophenyl octyl ether | 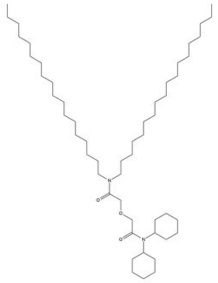 | Potassium tetrakis(4-chlorophenyl)borate |
| 6 | | | 29 | 66 | 5 | 0 |
| 7 | | | 31 | 64 | 4.5 | 0.5 |
| 8 | $Ca^{2+}$ | 100 | 33 | 62 | 4 | 1 |
| 9 | | | 35 | 60 | 3.5 | 1.5 |
| 10 | | | 37 | 58 | 3 | 2 |
| | | | **trivalent sensors** | | | |
| **No.** | **Target ion** | **Ionic radius** | **PVC ratio (%)** | **Plasticizer type/ratio (%)** | **Ionophore structure/ratio (%)** | **Lipophilic additives type/ratio (%)** |
| | | | | 2-Nitrophenyl octyl ether | 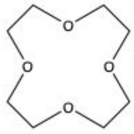 | Oleic acid |
| 1 | | | 26 | 59 | 5 | 10 |
| 2 | | | 28 | 57 | 6 | 9 |
| 3 | $La^{3+}$ | 103.2 | 30 | 55 | 7 | 8 |
| 4 | | | 32 | 53 | 8 | 7 |
| 5 | | | 34 | 51 | 9 | 6 |
| | | | | Dibutyl phthalate | 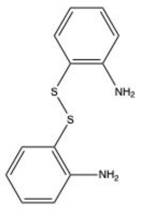 | Potassium tetrakis(4-chlorophenyl)borate |

| | | | | | | |
|---|---|---|---|---|---|---|
| 6 | | | 31 | 64 | 5 | 0 |
| 7 | | | 33 | 62 | 4.5 | 0.5 |
| 8 | $Tm^{3+}$ | 88 | 35 | 60 | 4 | 1 |
| 9 | | | 37 | 58 | 3.5 | 1.5 |
| 10 | | | 39 | 56 | 3 | 2 |

**Table S5.** Membrane components and ratios used for ISE sensor fabrication to validate the Bayesian optimization on the Nernst slope and to determine the optimal detection limit.

| No. | Predicted slope (mV/Dec) | PVC ratio (%) | Plasticizer type/ratio (%) | Ionophore structure/ratio (%) | Lipophilic additives type/ratio (%) | Experiment-al slope (mV/Dec) | Detection limit (M) |
|---|---|---|---|---|---|---|---|
| | | | | **$Na^+$ sensor** | | | |
| | | | | 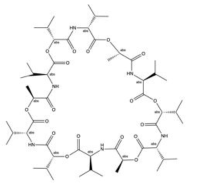 | | | |
| 1 | 59.14 | 33.5 | DPE: 52.7 | 3.8 | KBF4: 10 | 54.85 | $5.10\times10^{-7}$ |
| 2 | 59.13 | 36.9 | DPE: 42.5 | 5.6 | KBF4: 15 | 57.88 | $2.02\times10^{-6}$ |
| 3 | 59.19 | 31.9 | DPE: 55.4 | 3.1 | KBF4: 9.6 | 57.76 | $5.93\times10^{-6}$ |
| 4 | 59.20 | 37.9 | DPE: 39.1 | 7.1 | KBF4: 15.9 | 57.23 | $7.49\times10^{-6}$ |
| 5 | 59.08 | 30.9 | DPE: 56.1 | 2 | KBF4: 11 | 55.48 | $8.88\times10^{-6}$ |
| | | | | **$Mg^{2+}$ sensor** | | | |
| **No.** | **Predicted slope (mV/Dec)** | **PVC ratio (%)** | **Plasticizer type/ratio (%)** | **Ionophore structure/ratio (%)** 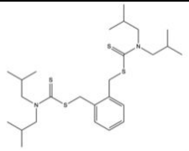 | **Lipophilic additives type/ratio (%)** | **Experiment al slope (mV/Dec)** | **Detection limit (M)** |
| 1 | 29.56 | 43.4 | BBPA: 27.4 | 3.8 | OA: 10 | 31.32 | $4.27\times10^{-7}$ |
| 2 | 29.57 | 56.5 | DPE: 33.6 | 5.6 | HMIMCl: 15 | 28.68 | $2.45\times10^{-6}$ |
| 3 | 29.61 | 41.3 | BBPA: 27.1 | 3.1 | OA: 9.6 | 28.92 | $1.80\times10^{-6}$ |
| | | | | 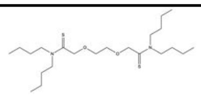 | | | |
| 4 | 29.59 | 49.6 | BES: 28.7 | 6.6 | HMIMCl: 21.9 | 27.70 | $9.58\times10^{-6}$ |
| 5 | 29.57 | 54.4 | DBP: 32 | 2.3 | HMIMCl: 15.1 | 28.74 | $4.01\times10^{-6}$ |
| | | | | **$Al^{3+}$ sensor** | | | |

| No. | Predicted slope (mV/Dec) | PVC ratio (%) | Plasticizer type/ratio (%) | Ionophore structure/ratio (%) 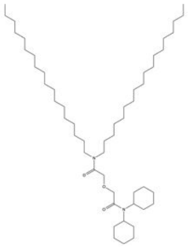 | Lipophilic additives type/ratio (%) | Experiment-al slope (mV/Dec) | Detection limit (M) |
|---|---|---|---|---|---|---|---|
| 1 | 19.71 | 66.3 | NPOE: 20.1 | 11 | BMIMCl: 2.6 | 19.50 | $1.21\times10^{-5}$ |
| 2 | 19.70 | 60.4 | NPOE: 29.3 | 8.1 | BMIMCl: 2.2 | 19.06 | $1.28\times10^{-6}$ |
| 3 | 19.69 | 66.2 | NPOE: 20.1 | 11.8 | BMIMCl: 1.9 | 18.42 | $7.36\times10^{-6}$ |
| 4 | 19.69 | 67 | NPOE: 20.3 | 10.2 | BMIMCl: 2.5 | 19.54 | $6.62\times10^{-7}$ |
| 5 | 19.70 | 63.8 | NPOE: 22.1 | 12 | BMIMCl: 2.1 | 18.11 | $4.36\times10^{-6}$ |

* **DPE:** Diphenyl ether, **KBF4:** Potassium tetrafluoroborate, **BBPA:** Bis(1-butylpentyl) adipate, **BES:** N,N-bis(2-hydroxyethyl)-2-aminoethanesulfonic acid, **DBP:** Dibutyl phthalate, **OA:** Oleic acid, **HMIMCl:** 1-Hexyl-3-methylimidazolium chloride, **NPOE:** 2-Nitrophenyl octyl ether, **BMIMCl:** 1-Butyl-3-methylimidazolium chloride.

**Table S6.** Evaluation of model performance in the detection limit

| Dataset | | training size | training $R^2$ | training RMSE | test size | test $R^2$ | test RMSE |
|---|---|---|---|---|---|---|---|
| Separated | monovalent sensor | 114 | 0.47 | $4.22\times10^{-5}$ | 29 | 0.29 | $2.54\times10^{-5}$ |
| | divalent sensor | 301 | 0.78 | $4.10\times10^{-4}$ | 76 | 0.10 | $9.70\times10^{-4}$ |
| | trivalent sensor | 144 | 0.51 | $7.13\times10^{-5}$ | 36 | 0.09 | $3.61\times10^{-5}$ |
| | together | 559 | 0.69 | $1.03\times10^{-4}$ | 141 | 0.29 | $4.9\times10^{-4}$ |

**Table S7.** The new candidate ionophores used for Bayesian optimization which are not included in the dataset.

| No. | Simplified Molecular Input Line Entry System (SMILES) |
|---|---|
| 1 | C[C@@H]1C[C@H]2CC[C@H](O2)[C@@H](C(=O)O[C@H](C[C@@H]3CC[C@@H](O3)[C@H](C(=O)O[C@@H](C[C@H]4CC[C@H](O4)[C@@H](C(=O)O[C@H](C[C@@H]5CC[C@@H](O5)[C@H](C(=O)O1)C)C)C)C)C)C)C |
| 2 | C[C@H]1C(=O)N[C@H](C(=O)O[C@@H](C(=O)N[C@@H](C(=O)O[C@H](C(=O)N[C@H](C(=O)O[C@@H](C(=O)N[C@@H](C(=O)O[C@H](C(=O)N[C@H](C(=O)O[C@@H](C(=O)N[C@@H](C(=O)O1)C(C)C)C(C)C)C(C)C)C)C(C)C)C(C)C)C(C)C)C)C(C)C)C(C)C)C(C)C |
| 3 | [H][C@@]1(O[C@@]2(CC[C@@H](C)[C@@H](Cc3nc4c(C(O)=O)c(NC)ccc4o3)O2)[C@H](C)C[C@H]1C)[C@H](C)C(=O)c5ccc[nH]5 |
| 4 | CCOC(=O)COc1c2Cc3cc(cc(Cc4cc(cc(Cc5cc(cc(Cc1cc(c2)C(C)(C)C)c5OCC(=O)OCC)C(C)(C)C)c4OCC(=O)OCC)C(C)(C)C)c3OCC(=O)OCC)C(C)(C)C |
| 5 | O=C(COc1ccccc1OCC(=O)N(Cc2ccccc2)c3ccccc3)N(Cc4ccccc4)c5ccccc5 |
| 6 | C1COC23CCCCC2(CCCC3)OCCOCCOC45CCCCC4(CCCC5)OC1 |
| 7 | CCCCCCCCCCCCCCCCCCN(CCCCCCCCCCCCCCCCCC)C(=O)COCC(=O)N(C1CCCCC1)C2CCCCC2 |
| 8 | CCCCCCCCCCCCC(C)(COC(=O)Nc1cc2OCCOCCOCCOCCOc2cc1[N+]([O-])=O)COC(=O)Nc3cc4OCCOCCOCCOCCOc4cc3[N+]([O-])=O |
| 9 | CCOC(=O)CCCCCCCCCCN(C)C(=O)CO[C@H](C)[C@@H](C)OCC(=O)N(C)CCCCCCCCCCCC(=O)OCC |
| 10 | CCCCNC(=S)Nc1cc(cc2c1Oc3c(NC(=S)NCCCC)cc(cc3C2(C)C)C(C)(C)C)C(C)(C)C |
| 11 | O=C(CC(=O)N1CCOCCOCCN(CCOCCOCC1)C(=O)CC(=O)NC23CC4CC(CC(C4)C2)C3)NC56CC7CC(CC(C7)C5)C6 |
| 12 | CCCCN(CCCC)C(=S)COCCOCC(=S)N(CCCC)CCCC |
| 13 | CCCCCCCCCCCCCCCCCCNC(=O)COCC(=O)N1CCOCCOCCOCCN(CCOCCOCC1)C(=O)COCC(=O)NCCCCCCCCCCCCCCCCCC |
| 14 | CCCCCCCN(C)C(=O)CC(=O)NCCCCCCCCNC(=O)CC(=O)N(C)CCCCCCC |
| 15 | CCC(COCC(=O)N(C1CCCCC1)C2CCCCC2)(COCC(=O)N(C3CCCCC3)C4CCCCC4)COCC(=O)N(C5CCCCC5)C6CCCCC6 |
| 16 | CCCCCCCN(C)C(=O)CCC(=O)N(C)CCCCCCC |
| 17 | CN(C(=O)CC(=O)NCCCCCc1cc(CCCCCNC(=O)CC(=O)N(C)C23CC4CC(CC(C4)C2)C3)cc(CCCCCNC(=O)CC(=O)N(C)C56CC7CC(CC(C7)C5)C6)c1)C89CC%10CC(CC(C%10)C8)C9 |
| 18 | C1COCCOCC(COCCOC1)(Cc2ccccc2)Cc3ccccc3 |
| 19 | CCCCC(CC)(COCC(=O)N(C1CCCCC1)C2CCCCC2)COCC(=O)N(C3CCCCC3)C4CCCCC4 |
| 20 | CNc1c(Br)cc2oc(C[C@H]3O[C@@]4(CC[C@H]3C)O[C@@H]([C@H](C)C[C@H]4C)[C@H](C)C(=O)c5ccc[nH]5)nc2c1C(O)=O |
| 21 | CC(C)CN(CC(C)C)C(=S)SCc1ccccc1CSC(=S)N(CC(C)C)CC(C)C |
| 22 | CNc1ccc2oc(C[C@H]3O[C@@]4(CC[C@H]3C)O[C@@H]([C@H](C)C[C@H]4C)[C@H](C)C(=O)c5ccc[nH]5)nc2c1C(=O)O[Ca]OC(=O)c6c(NC)ccc7oc(C[C@H]8O[C@@]9(CC[C@H]8C)O[C@@H]([C@H](C)C[C@H]9C)[C@H](C)C(=O)c%10ccc[nH]%10)nc67 |
| 23 | [C@H](NC(=O)[C@H](OC(=O)[C@@H](NC(=O)[C@H](C)OC(=O)[C@H](NC(=O)[C@H] |

**Table S8.** The 16 candidate ionophores with MF similarities to both reference MFs to execute Bayesian optimization.

| No. | Simplified Molecular Input Line Entry System (SMILES) | Commercially available? |
|---|---|---|
| 1 | C[C@H]1C(=O)N[C@H](C(=O)O[C@@H](C(=O)N[C@@H](C(=O)O[C@H](C(=O)N[C@H](C(=O)O[C@@H](C(=O)N[C@@H](C(=O)O[C@H](C(=O)N[C@H](C(=O)O[C@@H](C(=O)N[C@@H](C(=O)O1)C(C)C)C(C)C)C(C)C)C)C(C)C)C(C)C)C(C)C)C)C(C)C)C(C)C)C(C)C | Yes |
| 2 | CC(C)CN(CC(C)C)C(=S)SCc1ccccc1CSC(=S)N(CC(C)C)CC(C)C | Yes |
| 3 | CCCCCCCCCCCCCCCCCCNC(=O)COCC(=O)N1CCOCCOCCOCCN(CCOCCOCC1)C(=O)COCC(=O)NCCCCCCCCCCCCCCCCCC | Yes |
| 4 | CCCCCCCN(C)C(=O)CC(=O)NCCCCCCCCNC(=O)CC(=O)N(C)CCCCCCC | Yes |
| 5 | CCCCN(CCCC)C(=S)COCCOCC(=S)N(CCCC)CCCC | Yes |
| 6 | CCOC(=O)CCCCCCCCCCCN(C)C(=O)CO[C@H](C)[C@@H](C)OCC(=O)N(C)CCCCCCCCCCCC(=O)OCC | Yes |
| 7 | CN(C(=O)CC(=O)NCCCCCc1cc(CCCCCNC(=O)CC(=O)N(C)C23CC4CC(CC(C4)C2)C3)cc(CCCCCNC(=O)CC(=O)N(C)C56CC7CC(CC(C7)C5)C6)c1)C89CC%10CC(CC(C%10)C8)C9 | Yes |
| 8 | CNc1c(Br)cc2oc(C[C@H]3O[C@@]4(CC[C@H]3C)O[C@@H]([C@H](C)C[C@H]4C)[C@H](C)C(=O)c5ccc[nH]5)nc2c1C(O)=O | Yes |
| 9 | CNc1ccc2oc(C[C@H]3O[C@@]4(CC[C@H]3C)O[C@@H]([C@H](C)C[C@H]4C)[C@H](C)C(=O)c5ccc[nH]5)nc2c1C(=O)O[Ca]OC(=O)c6c(NC)ccc7oc(C[C@H]8O[C@@]9(CC[C@H]8C)O[C@@H]([C@H](C)C[C@H]9C)[C@H](C)C(=O)c%10ccc[nH]%10)nc67 | Yes |
| 10 | O=C(CC(=O)N1CCOCCOCCN(CCOCCOCC1)C(=O)CC(=O)NC23CC4CC(CC(C4)C2)C3)NC56CC7CC(CC(C7)C5)C6 | Yes |
| 11 | O=C(COc1ccccc1OCC(=O)N(Cc2ccccc2)c3ccccc3)N(Cc4ccccc4)c5ccccc5 | Yes |
| 12 | CCCCC(CC)(COCC(=O)N(C1CCCCC1)C2CCCCC2)COCC(=O)N(C3CCCCC3)C4CCCCC4 | Yes |
| 13 | [H][C@@]1(O[C@@]2(CC[C@@H](C)[C@@H](Cc3nc4c(C(O)=O)c(NC)ccc4o3)O2)[C@H](C)C[C@H]1C)[C@H](C)C(=O)c5ccc[nH]5 | Yes |
| 14 | CCOC(=O)COc1c2Cc3cc(cc(Cc4cc(cc(Cc5cc(cc(Cc1cc(c2)C(C)(C)C)c5OCC(=O)OCC)C(C)(C)C)c4OCC(=O)OCC)C(C)(C)C)c3OCC(=O)OCC)C(C)(C)C | No |
| 15 | C1COC23CCCCC2(CCCC3)OCCOCCOC45CCCCC4(CCCC5)OC1 | No |
| 16 | CCCCCCCCCCCCCCCCCCN(CCCCCCCCCCCCCCCCCC)C(=O)COCC(=O)N(C1CCCCC1)C2CCCCC2 | No |
